# A Study of the Relationship between Professional Communication and Professional Commitment in Operating Room Nurses

**DOI:** 10.1155/2022/5871846

**Published:** 2022-07-05

**Authors:** Hamed Parnikh, Camellia Torabizadeh, Majid Najafi Kalyani, Mitra Soltanian

**Affiliations:** ^1^Student Research Committee, Shiraz University of Medical Sciences, Shiraz, Iran; ^2^Department of Nursing, School of Nursing and Midwifery, Community Based Psychiatric Care Research Center, Shiraz University of Medical Sciences, Shiraz, Iran; ^3^Department of Medical Emergencies, School of Nursing and Midwifery, Shiraz University of Medical Sciences, Shiraz, Iran; ^4^Department of Nursing, School of Nursing and Midwifery, Shiraz University of Medical Sciences, Shiraz, Iran

## Abstract

**Background:**

Operating rooms are among the most complex clinical environments in hospitals where teamwork and professional communication play a very important part. Professional commitment is an influential factor in the personnel's professional communication and can affect the outcomes of healthcare services.

**Objective:**

The present study aims to investigate the relationship between professional communication and professional commitment in the operating room nurses in teaching hospitals. *Study Design*. The present study used a descriptive-correlational design. Participants were selected via census sampling from hospitals in Shiraz over a period of three months. The participants were 350 operating room nurses who met the inclusion criteria. Data were collected using professional communication and a professional commitment questionnaire. The collected data were analyzed using the descriptive and inferential statistics in SPSS *v*v. 22. *Findings*. The operating room nurses' mean scores for professional communication (136.81 ± 13.77) and professional commitment (71.42 ± 11.62) were found to be high. The results of the Pearson correlation coefficient test showed that there was a statistically significant linear relationship between professional communication and professional commitment (*p* < 0.001, *r* = 0.235). The results also showed that there was a significant relationship between professional commitment on the one hand and gender (*p*=0.042), marital status (*p*=0.047), and interest in work (*p*=0/000) on the other hand. There was also a significant relationship between professional communication and interest in work (*p*=0/000).

**Conclusion:**

Given the significance of professional communication and professional commitment in the operating rooms, it is recommended that healthcare administrators and policymakers take steps to improve these areas. It is suggested that measures should be taken to increase the personnel's awareness of the principles of professional communication and the significance of professional commitment through regular workshops.

## 1. Introduction

Because of its direct impact on human health, the healthcare system is viewed as one of the most important areas for continuing growth in all societies [1]. Operating rooms are among the most complex clinical environments in healthcare organizations [1]. All the members of a surgical team, including operating room nurses, nurse anesthesiologists, and doctors, must communicate with each other effectively to provide safe care to patients [2]. As the operating rooms are very stressful and challenging environments, satisfactory professional communication between nurses and doctors is essential for providing satisfactory care and maintaining patient safety [3].

Poor professional communication between operating room nurses and doctors results in stress, inability to concentrate, anger, lack of cooperation, distrust, decline in the quality of care, disregard for safety, and even death of patients [4, 5]. Studies show that poor communication among the operating room personnel due to verbal conflicts, interpersonal issues, unprofessional relationships, stress, and work overload can lead to patient dissatisfaction, retained surgical items, and operation on the wrong organ [6, 7]. In addition, if the members of the surgical team (the operating room personnel, nurse anesthesiologists, surgeons, and anesthesiologists) do not play their part in sharing information, the risk of death and postoperative complications increases up to four times [8].

Interpersonal communication and cooperation in hospitals are essential for achieving success. Satisfactory cooperation between the personnel improves the performance of healthcare teams and results in better patient satisfaction [9]. However, in different departments of hospitals, occasional conflicts occur between healthcare professionals. Conflicts between nurses and doctors are known to be an inevitable part of professional relationships in hospitals with potentially adverse effects on the quality of care provided to patients [10]. Effective communication correlates with job satisfaction, motivation, professional performance, productivity, organizational atmosphere, leadership strategies, and organizational commitment [11].

Professional commitment, on the other hand, is an influential factor in the personnel's professional communication and affects the outcome of healthcare services [6, 7]. Professional commitment is defined as a sense of identity and attachment to a particular profession and willingness to work at a job [12]. Professional commitment is an important predicting factor in nurses' professional performance [8] and a facilitating factor in reducing nurses' emotional fatigue due to work overload [13]. Absenteeism, job burnout, and medical errors have been reported to be more common among nurses with lower levels of professional commitment [14]. On the other hand, high professional commitment in the healthcare personnel results in better job satisfaction [11]. Professional commitment also helps healthcare organizations achieve their objectives, namely, providing adequate quality healthcare services to patients and increasing patient safety and satisfaction [15].

Despite the significance of the concepts of professional communication and professional commitment in clinical environments, an extensive review of the literature showed that no studies had specifically addressed the relationship between professional communication and professional commitment in operating room nurses. Accordingly, the present study was conducted to investigate the relationship between professional communication and professional commitment in this population.

## 2. Method

### 2.1. Study Design and Sampling

The present study is a descriptive-correlational work of research. The sample was selected via census sampling from 7 teaching hospitals located in Shiraz, the largest city in the south of Iran. After acquiring the phone numbers and email addresses of the subjects from the main office of nurses in the province, the researchers contacted them and asked them to participate in the study. In total, 350 operating room nurses who met the inclusion criteria participated in this study. The inclusion criteria were being willing to participate, having at least an associate degree in operating room nursing or anesthesia, and having a minimum of six months of experience of practice in operating rooms. The subjects who were not willing to participate or failed to complete the questionnaires fully were excluded. Because of the COVID-19 pandemic, the questionnaires were converted to online surveys using Porsline and then sent to the participants via the social networks such asWhatsApp and Telegram.

### 2.2. Data Collection Instruments

The data collection instruments consisted of a demographics survey (age, gender, marital status, education, work experience, specialty, interest in work, and work shift), a professional communication questionnaire, and a professional commitment questionnaire. Developed by Torabizadeh et al. in 2019, the operating room professional communication questionnaire was reported to have satisfactory psychometric properties. The questionnaire consists of 41 items in six domains: mutual respect and trust, teamwork, ethical competence, physical conditions of the work environment, workplace conflicts, and interprofessional interactions. Scoring is on a 5-point Likert scale, ranging from never to always (never = 1; rarely = 2; occasionally = 3; often = 4; always = 5). 13 items are reverse scored. The score range of the questionnaire is between 93 and 153. The respondents' professional communication scores are divided into three categories: poor (93–113), average (113–133), and strong (133–153). The validity of the questionnaire has been measured in terms of its face validity, content validity, and construct validity. Measured by the internal homogeneity and consistency methods, the reliability of the questionnaire has been calculated to be a Cronbach's alpha of 0.92. The internal homogeneity of the instrument has also been measured by calculation of its Cronbach's alpha, whose value was 0.7 to 0.8. The consistency of the questionnaire was evaluated using the test-retest method [16].

The professional commitment questionnaire was developed by Lin et al. in 2007. The questionnaire consists of 19 items that address the three domains of satisfaction with the nursing profession, involvement in the nursing profession, and staying in the nursing profession. Responses to the items are arranged on a 5-point Likert scale, ranging from “I completely disagree = 1” to “I completely agree = 5.” The validity and reliability of the instrument have been assessed and verified by the creators of the instrument. The Cronbach's alpha coefficient of total scores was 0.91, implying that the instrument was internally and consistently structured. The test_retest reliability of total scores was 0.91, suggesting that the NPCS instrument was consistent over time. The score range of the questionnaire is between 19 to95. Professional commitment scores fall into three categories: poor (19–44.33), average (44.3,3–69.66), and strong (69.66–95) [17].

### 2.3. Data Analysis

The collected data were analyzed in the IBM SPSS *v*v. 22. The quantitative variables were reported using mean and standard deviation, and the qualitative variables were reported in frequency and percentage. The data were analyzed using the statistical tests of the Pearson correlation coefficient, Spearman correlation coefficient, independent *t*-test, and one-way ANOVA. The significance level was set at*p* < 0.05.

### 2.4. Ethical Considerations

The present study was conducted after it had been approved by the ethics committee at the Shiraz University of Medical Sciences (ethics code: IR.SUMS.REC.1400.078). To preserve the participants' anonymity and the confidentiality of their information, the questionnaires were assigned codes before being submitted to the nurses. The participants were informed about the objectives of the study and the voluntary nature of their participation. All the nurses who participated in the study had completed the informed consent form.

## 3. Findings

Of the 350 operating room nurses who participated, the majority were female (61.4%), were married (60%), and had a bachelor's degree (83.1%). The participants' mean age and work experience were 32.27 ± 7.17 and 8.93 ± 7.08 years, respectively (Table 1).

The results showed that the operating room nurses' mean scores for professional communication (136.81 ± 13.77) and professional commitment (71.42 ± 11.62) were high. The highest professional communication means score was related to the dimension of interprofessional interactions (32.96 ± 5.66) and the highest professional commitment mean score was related to the dimension of involvement in one's profession (37.46 ± 4.81) (Table 2).

The results of the Pearson correlation coefficient test showed that there was a statistically significant direct relationship between the operating room nurses' professional communication and professional commitment mean scores (*p* < 0.001, *r* = 0.235), meaning that an increase in their professional communication scores correlated with an increase in their professional commitment scores and vice versa. It was also found that there was a direct one-to-one relationship between many dimensions of the variables, except for the dimensions of involvement in the profession, staying in the profession, workplace conflicts, and interprofessional interactions (Table 3).

The results of the study showed that there was a statistically significant relationship between the mean scores for professional communication and interest in work. On the other hand, the relationship between the participants' professional communication mean scores and the variables of age, gender, marital status, education, academic major, work experience, and work shift was not significant (*p* > 0.05) (Table 4).

The relationship between the participants' professional commitment mean scores on the one hand and their gender (*p*=0.041), marital status (*p*=0.047), and interest in work on the other was found to be statistically significant. However, the relationship between the professional commitment mean scores and the variables of age, education, academic major, work experience, and work shift was not significant (*p* > 0.05) (Table 4).

## 4. Discussion

The present study investigated the relationship between professional communication and professional commitment, as well as the relationship between these two variables and demographic variables in operating room nurses. Overall, the results showed that the participants' professional communication and professional commitment mean scores were high and that there was a significant direct relationship between the two concepts.

In the present study, the operating room nurses' professional communication mean score was high. Similarly, studying professional communication between doctors and nurses and its impact on the quality of patient care, Ghahramanian et al. reported that professional communication between doctors and nurses was satisfactory and that this improved the quality of care provided to patients [12]. Also, the results of a study by Song et al. showed that competence in professional communication enables special care nurses to provide better quality care to their patients [18]. These research findings are consistent with the results of the present study. On the other hand, the results of a study in South Africa showed that the nurses' satisfaction with their interprofessional communication was low [19]. The discrepancy can be attributed to the fact that the present study evaluated communication between operating room nurses and doctors, but the abovementioned study measured nurses' satisfaction with their communication with doctors and the other hospital staff. Also, the sample size of the present study was larger.

One of the dimensions of professional communication addressed in the present study was interprofessional interactions, which attracted the highest score. The results of another study shows that improvement in interprofessional interactions between nurses and doctors results in better professional performance and less errors [20]. According to another study conducted in Palestine, almost 75% of nurses and 24% of doctors have a positive perception of their interprofessional communication [21]. The results of a study by Amudha et al. shows that nurses' poor practical competence, barriers in the workplace, and doctors' characteristics are the three main factors in the communication gap between doctors and nurses. Effective communication among healthcare providers is crucial to the success of every healthcare system and can guarantee good decision-making surrounding patients' care. Communication and teamwork are the backbone of healthcare organizations and contribute to patients' safety [22]. In contrast to the findings of the present study, the results of a similar study show that operating room nurses perceive their communication with doctors to be unsatisfactory because of the latter's domineering attitude [23]. Discrepancies between the nurses' perceptions may be due to differences between cultural and religious contexts, data collection instruments, and research settings.

In the present study, the operating room nurses perceived the status of professional commitment to be strong. The highest mean score in this domain was related to the dimension of involvement in the profession. Similarly, the results of a study in Taiwan show nurses' professional commitment to be satisfactory [24]. Also, a cross-sectional study of 384 nurses conducted in Turkey shows the level of nurses' professional commitment to be high [25]. In a study by Hsu et al., nurses' professional commitment was found to be satisfactory. Also, as in the present study, the highest professional commitment mean score was for involvement in a profession [26].

Another finding of the present study is that there is a significant positive relationship between operating room nurses' professional communication and professional commitment. Thus, an increase in one index correlates with an increase in the other and vice versa. Studies in which these two variables have been explored independently show that professional communication and commitment both have a significant direct relationship with nurses' job satisfaction [27, 28]. Moreover, each index can separately contribute to nurses' self-efficacy [28].

The findings of the present study showed that the relationship between the participants' mean age, gender, academic major, work experience, education, and work shift on the one hand and their professional communication mean score on the other hand was not significant. However, the nurses who had greater interest in their academic major achieved higher scores in the domain of professional communication. The results of a cross-sectional study in Iran show that there is a significant positive relationship between nurses' professional communication and interest in work, which is consistent with the findings of the present study [29].

In the present study, the relationship between the participant's age, work experience, education, and work shift and their professional commitment mean score was not significant. However, female and married nurses who were interested in their profession achieved higher professional commitment scores. In their study, Mersin et al. report a significant positive relationship between professional commitment and interest in a profession [30]. Likewise, the results of the present study showed that the participants' professional commitment correlated positively with their interest in their profession. Also, according to a study by Hsu et al., there is a significant relationship between professional commitment on the one hand and marital status and employment status on the other hand, which finding is consistent with the results of the present study [26].

According to a cross-sectional study of nurses in Turkey, there is a significant positive relationship between professional commitment and age and work shift in nurses who work fixed shifts and are aged over 40 years, which finding is not consistent with the results of the present study [31]. The discrepancy may be due to cultural and organizational differences between the two study populations. It is suggested that future studies in this area address the possible relationship between professional commitment and cultural and social factors.

## 5. Conclusion

Professional communication and commitment in the personnel in operating rooms can guarantee patient safety and high-quality care. The findings of the present study show that there is a significant direct relationship between professional communication and professional commitment in the operating room personnel. Today's healthcare systems need committed personnel who are capable of effective professional communication even when complex ethical conflicts are present. Accordingly, operating room personnel's awareness of these concepts should be raised, and policymakers should take measures to improve professional communication and commitment in this population.

### 5.1. Study Limitations

The concurrence of the present study with the COVID-19 pandemic limited the researchers' access to the subjects of the study. Accordingly, the questionnaires were developed using Porsline and sent to the participants. Even though the questionnaires were completed virtually, some of the participants may have provided inaccurate answers, which could have affected the results of the study.

## Figures and Tables

**Table 1:** The participants' demographic characteristics.

Variable		Number (percentage)
Gender	Male	135 (38.6)
	Female	215 (61.4)
Education	Associate degree	45 (12.9)
	Bachelor's degree	291 (83.1)
	Master's degree	14 (4)
Marital status	Single	140 (40)
	Married	210 (60)
Academic major	Operating room	253 (72.3)
	Anesthesia	97 (27.7)
Work shift	Fixed	65 (18.6)
	Rotational	285 (81.4)
Interest in work	Yes	301 (86)
	No	49 (14)

**Table 2:** The means and standard deviations of the participants' professional communication and professional commitment scores.

Dimensions of professional communication	Mean ± SD	Dimensions of professional commitment	Mean ± SD
Mutual respect and trust	74.38 ± 2.14	Satisfaction with profession	24.06 ± 5.65
Teamwork	13.66 ± 2.71	Involvement in profession	37.46 ± 4.81
Ethical competence	31.39 ± 5.66	Staying in profession	9.89 ± 3.03
Physical conditions of the work environment	23.26 ± 3.3	Total score	71.41 ± 11.61
Workplace conflicts	21.14 ± 2.84		
Interprofessional interactions	32.96 ± 2.84		
Total score	136.80 ± 13.76		

**Table 3:** The relationships between the dimensions of professional communication and professional commitment.

Professional communication total score	Dimensions of professional communication						Variable	Variable
	Interprofessional interactions	Workplace conflicts	Physical conditions of the work environment	Ethical competence	Teamwork	Respect		
136.81±13.77	*r* = 0.048 *p*=0.369	*r* = 021 *p*=0.691	*r* = 0.157 *p*=0.03^*∗*^	*r* = 0.297 *p*=0.000^*∗*^	*r* = 0.244 *p*=0.000^*∗*^	*r* = 0.186 *p*=0.000^*∗*^	Satisfaction	Dimensions of professional commitment communication
	*r* = 0.056 *p*=0.300	*r* = 0.077 *p*=0.151	*r* = 0.122 *p*=0.023^*∗*^	*r* = 0.250 *p*=0.000^*∗*^	*r* = 0.255 *p*=0.000^*∗*^	*r* = 0.192 *p*=0.000^*∗*^	Involvement in profession	
	*r* = 0.022 *p*=0.676	*r* = 0.047 *p*=0.378	*r* = 0.121 *p*=0.024^*∗*^	*r* = 0.245 *p*=0.000^*∗*^	*r* = 0.195 *p*=0.000^*∗*^	*r* = 0.138 *p* = 0.010^*∗*^	Staying in profession	
*r* = 0.235 *p*=>0.001^*∗*^	71.42 ± 11.62						Professional commitment total score	

^*∗*^The relationship is significant (*p* value<0.05).

**Table 4:** The relationship between the participants' professional communication and professional commitment mean scores and their demographic characteristics.

Demographic characteristics		Variable	
		Professional commitment	Professional communication
Age^a^		*r* = 0.016; *p*=0.186	*r* = 0.066; *p*=0.216
Work experience^a^		*r* = 0.056; *p*=0.281	*r* = 0.065; *p*=0.203
Academic major^b^	Operating room	72.21 ± 10.37	137.05 ± 13.37
	Anesthesia	69.32 ± 14.22	136.14 ± 14.79
	*p* value	0.071	0.518
Gender^c^	Male	68.3481 ± 12.11	135.6 ± 130.8
	Female	73.3442 ± 10.8	137.5302 ± 13.3
	*p* value	0.041^*∗*^	0.665
Work shift^c^	Fixed	71.9 ± 11.8	140.0 ± 11.31
	Rotational	71.29 ± 11.7	141.53 ± 11.41
	*p* value	0.672	0.155
Marital status^c^	Single	69.8 ± 12.9	135.7786 ± 13.08
	Married	72.4 ± 10.5	137.4905 ± 14.045
	*p* value	0.047^*∗*^	0.255
Interest in work^c^	Yes	73.5 ± 9.8	137.87 ± 13.12
	No	58.3 ± 10.5	130.2 ± 14.81
	*p* value	0.000^*∗*^	0.000^*∗*^
Education^b^	Associate degree	73.4000 ± 10.42593	139.2 0 ± 12.31
	Bachelor's degree	71.0481 ± 11.7	140.0 ± 11.93
	Master's degree	72.7 ± 11.5	139.64 ± 13.02
	*p* value	0.412	0.634

^*∗*^The relationship was significant (*p* < 0.05). ^*∗*^^a^Pearson correlation test. ^b^ANOVA. ^c^*t*-test.

## Data Availability

The data used to support the findings of this study are available from the corresponding author upon request.
